# Intracellular NAD ^+^ Depletion Increases Prostanoid Production via p38/COX2 Signalling in FK866‐Induced Senescent Human Umbilical Vein Endothelial Cells

**DOI:** 10.1111/jcmm.71187

**Published:** 2026-05-20

**Authors:** Natsuko Kitajima, Takahisa Nakajo, Takeshi Katayoshi, Kentaro Tsuji

**Affiliations:** ^1^ DHC Corporation Laboratories Chiba Japan

**Keywords:** cardiovascular disease, human umbilical vein endothelial cell, nicotinamide adenine dinucleotide, prostanoid

## Abstract

Vascular endothelial cells maintain vascular homeostasis by releasing mediators with vasoconstrictive and vasorelaxant effects. Prostanoids are bioactive substances synthesised by cyclooxygenase2 (COX2); they preserve vascular function and can increase the risk of cardiovascular disease (CVD) associated with ageing. Age‐related reductions in nicotinamide adenine dinucleotide (NAD^+^) have been implicated in CVD pathogenesis. However, the relationship between intracellular NAD^+^ levels and prostanoid production in vascular endothelial cells is unclear. Herein, reduced intracellular NAD^+^ levels upregulated COX2 protein expression by activating the p38 mitogen‐activated protein kinase (MAPK) signalling pathway, increasing the production of prostaglandin F_1α_ (PGF_1α_) and thromboxane B_2_ (TXB_2_) in human umbilical vein endothelial cells (HUVECs). Treatment with the nicotinamide phosphoribosyltransferase (NAMPT) inhibitor, FK866, decreased intracellular NAD^+^ levels, induced a cellular senescence‐like phenotype characterised by the suppression of cell proliferation without cell death and promoted PGF_1α_ and TXB_2_ production in HUVECs. FK866 treatment increased phosphorylated p38 and COX2 protein levels, whereas treatment with the p38 inhibitor, PD169316, suppressed the FK866‐induced increase in COX2 expression. Supplementation with nicotinamide mononucleotide (NMN), a precursor of NAD^+^, following FK866 treatment restored intracellular NAD^+^ levels, reduced the cellular senescence‐like phenotype and attenuated the production of PGF_1α_ and TXB_2_. These results suggest that intracellular NAD^+^ appears to regulate prostanoid production in HUVECs under conditions of acute depletion.

## 1 Introduction

Vascular endothelial cells, which constitute the innermost layer of blood vessels throughout the body, play a crucial role in maintaining vascular function. In addition to serving as a barrier between the bloodstream and surrounding tissues, endothelial cells synthesise and release several vasoactive mediators that regulate vascular tone and homeostasis [1]. Prostanoids are physiologically active lipid mediators derived from the arachidonic acid cascade. Arachidonic acid, released from membrane phospholipids, is converted by cyclooxygenase (COX) enzymes and specific synthases into prostaglandin E_2_ (PGE_2_), PGD_2_, PGF_2α_, PGI_2_ and thromboxane A_2_ (TXA_2_). Among these, PGI_2_ and TXA_2_ exert vasodilatory and vasoconstrictive effects, respectively, and are regarded as important prostanoids in the cardiovascular system [2]. COX enzymes are classified into two isoforms, COX1, which is constitutively expressed in most tissues, and COX2, which is typically absent under normal conditions but is inducible by inflammatory stimuli, such as cytokines and growth factors [3, 4].

Cardiovascular disease (CVD) is the leading cause of mortality worldwide, with CVD incidences significantly increasing with age [5]. Endothelial dysfunction is a hallmark of many age‐associated cardiovascular conditions, including atherosclerosis, hypertension, diabetes and heart failure [6]. Previous studies have reported that prostanoid‐producing enzymes and their receptors are overexpressed in aged vascular endothelial and smooth muscle cells [7]. Since prostanoids contribute to the pathogenesis of CVD [2], age‐related dysregulation in their production may represent a mechanistic link to increased CVD risk [8].

The mitogen‐activated protein kinase (MAPK) cascade is an important intracellular signalling pathway that regulates a wide range of cellular processes. In mammalian cells, three major MAPK pathways, extracellular signal‐regulated kinase (ERK), p38 MAPK and c‐Jun N‐terminal kinase (JNK), are involved in distinct physiological responses [9]. These responses include regulation of the cell cycle, immune responses and inflammation. Dysregulation of MAPK signalling, which can be triggered by growth factors or environmental stress, has been implicated in the pathogenesis of various diseases, including CVD and neurodegenerative disorders. In fact, MAPK signalling is suggested to modulate prostaglandin synthesis in vascular tissues [10]. Studies exploring the role of p38 MAPK in CVD models, including atherosclerosis and myocardial infarction, have demonstrated that the oral administration of p38 inhibitors confers cardiovascular benefits [11]. Collectively, these findings suggest that p38 MAPK may contribute to endothelial dysfunction during ageing and merit further investigation.

Nicotinamide adenine dinucleotide (NAD^+^) is an important coenzyme that participates in redox reactions across numerous metabolic pathways, including glycolysis, the tricarboxylic acid cycle and the mitochondrial electron transport chain. Moreover, it serves as a substrate for several signalling enzymes. Evidence from animal and human studies indicates that intracellular NAD^+^ levels decline with ageing [12, 13, 14, 15]. This reduction is thought to contribute to age‐related pathophysiology by decreasing energy metabolism and the activity of NAD^+^‐dependent enzymes [16, 17]. Given that NAD^+^ cannot be directly absorbed from the external environment, it is synthesised endogenously via three major pathways: the salvage, Preiss–Handler and de novo pathways. Among these, the salvage pathway, which recycles nicotinamide (NAM) generated from NAD^+^ breakdown by enzymes such as sirtuins and poly‐adenosine diphosphate (ADP)‐ribose polymerase, is particularly important in mammals. NAM is converted into nicotinamide mononucleotide (NMN) by the rate‐limiting enzyme nicotinamide phosphoribosyltransferase (NAMPT). Subsequently, NMN is converted to NAD^+^ by NMN adenylyltransferase. NAMPT expression decreases with ageing in several tissues, such as the aorta, cerebral vessels [18] and cerebellum [19, 20], and this NAMPT reduction is responsible for the decrease in NAD^+^ associated with ageing.

From a vascular standpoint, the significance of NAD^+^ has been demonstrated: NAD^+^ supplementation in endothelial cells can prevent age‐related declines in angiogenesis and blood flow [20] and attenuates myocardial ischaemia/reperfusion‐induced microvascular damage [21]. Based on these findings, we hypothesised that NAD^+^ depletion may contribute to vascular dysfunction during ageing. Despite increasing interest in the relationship between vascular function and NAD^+^ metabolism, there have been few reports to date. Therefore, in this study, we aimed to investigate the effects of reduced intracellular NAD^+^ levels on prostanoid production and its underlying mechanisms in vascular endothelial cells.

## 2 Materials and Methods

### 2.1 Reagents

FK866 was purchased from AdooQ Bioscience (Irvine, CA, USA). Celecoxib was obtained from Tokyo Chemical Industry (Tokyo, Japan). PD169316 and the anti‐Lamin B1 antibody (#B‐10, 1:5000) were purchased from Santa Cruz Biotechnology (Dallas, TX, USA). NMN was purchased from Oriental Yeast (Tokyo, Japan). The anti‐β‐actin antibody (#ab8226, 1:5000) was obtained from Abcam (Cambridge, UK). Antibodies against COX2 (#12282, 1:5000), phospho‐p38 (#9211, 1:1000) and total p38 (#9212, 1:5000), as well as horseradish peroxidase (HRP)‐conjugated anti‐rabbit IgG (#7074) antibodies, were purchased from Cell Signalling Technology (Danvers, MA, USA). HRP‐conjugated anti‐mouse IgG (NA931) was purchased from GE Healthcare Biosciences (Chicago, IL, USA). All other reagents were of the highest commercially available grade.

### 2.2 Cell Cultures

HUVECs were purchased from Lonza (Basel, Switzerland) and cultured in KBM VEC‐1 basal medium (Kohjin Bio, Saitama, Japan) supplemented with 2% KBM VEC‐1 foetal bovine serum and KBM VEC‐1 supplement (Kohjin Bio). The cells were maintained in a humidified incubator at 37°C in 5% CO_2_. HUVECs were subcultured at 80%–90% confluence and used only until the sixth passage. For experiments, the cells were seeded at a density of 1.25 × 10^4^ cells/well in 12‐well plates and treated with each reagent 24 h after seeding. These commercially available cells were collected from donors with informed consent under the supplier's ethical approval. No further ethics approval was required.

### 2.3 Total NAD ^+^ Quantification

Intracellular NAD^+^ levels were measured using the NAD^+^/NADH Assay Kit‐WST (Dojindo Laboratories, Kumamoto, Japan) according to the manufacturer's instructions. HUVECs were lysed in NAD^+^/NADH Extraction Buffer, and the lysates were filtered using an Ultracel‐10 K centrifugal filter device (Merck, Darmstadt, Germany). For NADH‐specific measurement, a portion of the filtrate was incubated at 60°C for 1 h. All samples were subjected to enzymatic reactions at 37°C for 1 h, and absorbance was measured at 450 nm using an Infinite 200 Pro plate reader (Tecan, Männedorf, Switzerland). Protein concentrations were determined using the bicinchoninic acid (BCA) method. Total NAD^+^ levels were calculated by subtracting the NADH concentration from the total NAD^+^ plus NADH values.

### 2.4 Quantitative Polymerase Chain Reaction (qPCR)

Total RNA was isolated from cultured cells using an RNeasy Mini Kit (QIAGEN, Mississauga, Canada), according to the manufacturer's instructions, and RNA concentrations were measured using a NanoDrop Oneᶜ (Thermo Fisher Scientific, Waltham, MA, USA). The isolated RNA was stored in ribonuclease‐free water at—80°C. We synthesised the first‐strand cDNA from total RNA using a PrumeScript II first‐strand cDNA Synthesis Kit (Takara Bio, Shiga, Japan), according to the manufacturer's instructions. Target mRNA expression levels were measured using a QuantStudio 3 Real‐Time PCR System (Thermo Fisher Scientific) following the PrimeTime Std qPCR Assay (Integrated DNA Technologies, Coralville, Iowa, USA): *GAPDH* (assay ID: Hs.PT.39a.22214836), *p21* (assay ID: Hs.PT.58.40874346.g) and *p16* (assay ID: Hs.PT.58.14776964). All reactions were performed in triplicate. The housekeeping gene, *GAPDH*, was used for normalisation. The comparative C_T_ method was used to calculate the relative amounts of mRNA.

### 2.5 Western Blot Analysis

HUVECs were lysed in lysis buffer (20 mM Tris–HCl [pH 7.5], 150 mM NaCl, 5 mM calcium disodium edetate and 1% Triton‐X 100) containing a protease inhibitor cocktail (Roche Diagnostics, Basel, Switzerland) and a phosphatase inhibitor cocktail (Thermo Fisher Scientific). Lysates were centrifuged at 9168 × *g* for 5 min at 4°C, and the resulting supernatants were mixed with sample buffer (250 mM Tris–HCl [pH 6.8], 20% 2‐mercaptoethanol, 8% sodium dodecyl sulphate, 20% sucrose and 40 μg/μL bromophenol blue). Samples were separated by SDS‐PAGE using 10% polyacrylamide gels and transferred onto polyvinylidene difluoride membranes using a semi‐dry transfer system (Bio‐Rad Laboratories, Hercules, CA, USA) for 1 h at 100 mV. Membranes were blocked in 5% nonfat dry milk prepared in Tris‐buffered saline containing 0.1% Tween‐20 (TBS‐T) and then washed three times with TBS‐T. Membranes were incubated with primary antibodies overnight at 4°C. After washing three times with TBS‐T, membranes were incubated with HRP‐conjugated secondary antibodies at room temperature for 2 h. Protein bands were visualised using ImmunoStar LD detection reagent (Wako, Tokyo, Japan) according to the manufacturer's protocol. Band intensities were quantified using ImageJ software (National Institutes of Health, Bethesda, MD, USA).

### 2.6 Senescence‐Associated β‐Galactosidase (SA‐β‐Gal) Assay

SA‐β‐gal staining was performed using the Senescence Detection Kit (Abcam) according to the manufacturer's instructions. HUVECs were fixed in 0.4 mL of Fixative Solution for 15 min. After two washes with phosphate‐buffered saline (PBS), cells were incubated in 0.4 mL SA‐β‐gal staining solution at 37°C for 4 days. Stained cells were imaged using a Leica DM IL microscope (Leica, Wetzlar, Germany) in PBS. The percentage of SA‐β‐gal‐positive cells was quantified using ImageJ software.

### 2.7 5‐Ethynyl‐2′‐Deoxyuridine (EdU) Incorporation Assay

S‐phase DNA synthesis was assessed using a Click‐iT EdU Flow Cytometry Assay Kit (Thermo Fisher Scientific). Briefly, HUVECs were incubated with EdU at a final concentration of 10 μM for 2 h. Following EdU incorporation, cells were harvested and washed with PBS. Cells were fixed with 4% paraformaldehyde for 15 min at room temperature. Fixed cells were permeabilised using a saponin‐based permeabilisation buffer and subjected to the Click‐iT reaction according to the manufacturer's instructions. Incorporated EdU was analysed using a BD FACSLyric Flow Cytometer (Becton, Dickinson and Company, Franklin Lakes, NJ, USA). EdU fluorescence was detected using a 488 nm excitation laser and a 527/32 nm emission filter. Data were analysed using FlowJo software, and EdU‐positive cells were defined as cells undergoing active DNA synthesis (S phase).

### 2.8 PE‐Annexin V and 7‐AAD Staining Apoptosis Assay

Apoptotic and necrotic cell populations were quantified using PE‐Annexin V and 7‐AAD staining. Cells were seeded at a density of 2.5 × 10^4^ cells/well in a 6‐well plate and treated with 2.5 nM FK866 for 48 h. As a positive control for apoptosis, cells were treated with 1 μM actinomycin for 24 h. After treatment, cells were collected, washed with PBS and resuspended in 100 μL of 1× binding buffer. Cells were incubated with 5 μL PE‐Annexin V and 5 μL 7‐AAD for 15 min at room temperature in the dark (Becton, Dickinson and Company). After incubation, 400 μL of 1× binding buffer was added, and samples were analysed using a BD FACSLyric Flow Cytometer (Becton, Dickinson and Company) within 1 h. PE‐Annexin V or 7‐AAD fluorescence was detected using a 488 nm excitation laser and a 586/42 nm emission filter or a 488 nm excitation laser and a 700/54 nm emission filter respectively. Data were analysed with FlowJo software. Cell populations were classified as necrotic (Annexin V^−^/7‐AAD^+^) populations, late apoptotic (Annexin V^+^/7‐AAD^+^), early apoptotic (Annexin V^+^/7‐AAD^−^) and viable (Annexin V^−^/7‐AAD^−^).

### 2.9 Enzyme‐Linked Immunosorbent Assay (ELISA)

The concentrations of PGF_1α_ and TXB_2_ in the supernatants of cultured HUVECs were measured using the 6‐keto‐PGF_1α_ and TXB_2_ ELISA Kits (Abcam), respectively, according to the manufacturer's instructions. Culture supernatants were normalised to protein concentration and dispensed into PGF_1α_ or TXB_2_ assay plate wells, precoated with donkey anti‐sheep IgG antibody or goat anti‐rabbit IgG antibody respectively. Samples were incubated with alkaline phosphatase‐conjugated PGF_1α_ or TXB_2_ antigen and polyclonal antibodies specific to PGF_1α_ or TXB_2_ at room temperature on a mini‐shaker (Biosan SIA, Riga, Latvia) for 2 h at 420 rpm. After three washes, pNpp substrate was added, and plates were incubated at room temperature for 45 min. Absorbance was measured at 405 nm with a reference wavelength of 580 nm using an Infinite 200 Pro plate reader (Tecan).

### 2.10 Statistical Analysis

Data are expressed as the mean ± standard error from at least three independent biological experiments. Statistical significance was evaluated using the Student's *t‐*test for comparisons between two groups and one‐way analysis of variance followed by Tukey–Kramer post hoc testing for multiple group comparisons involving three or more groups, performed using Statcel3 software (OMS, Tokyo, Japan). A *p*‐value of less than 0.05 was considered statistically significant.

## 3 Results

### 3.1 FK866 Treatment Decreases Intracellular NAD ^+^ Levels and Induces Senescence‐Like Phenotype in HUVECs

**FIGURE 1:**
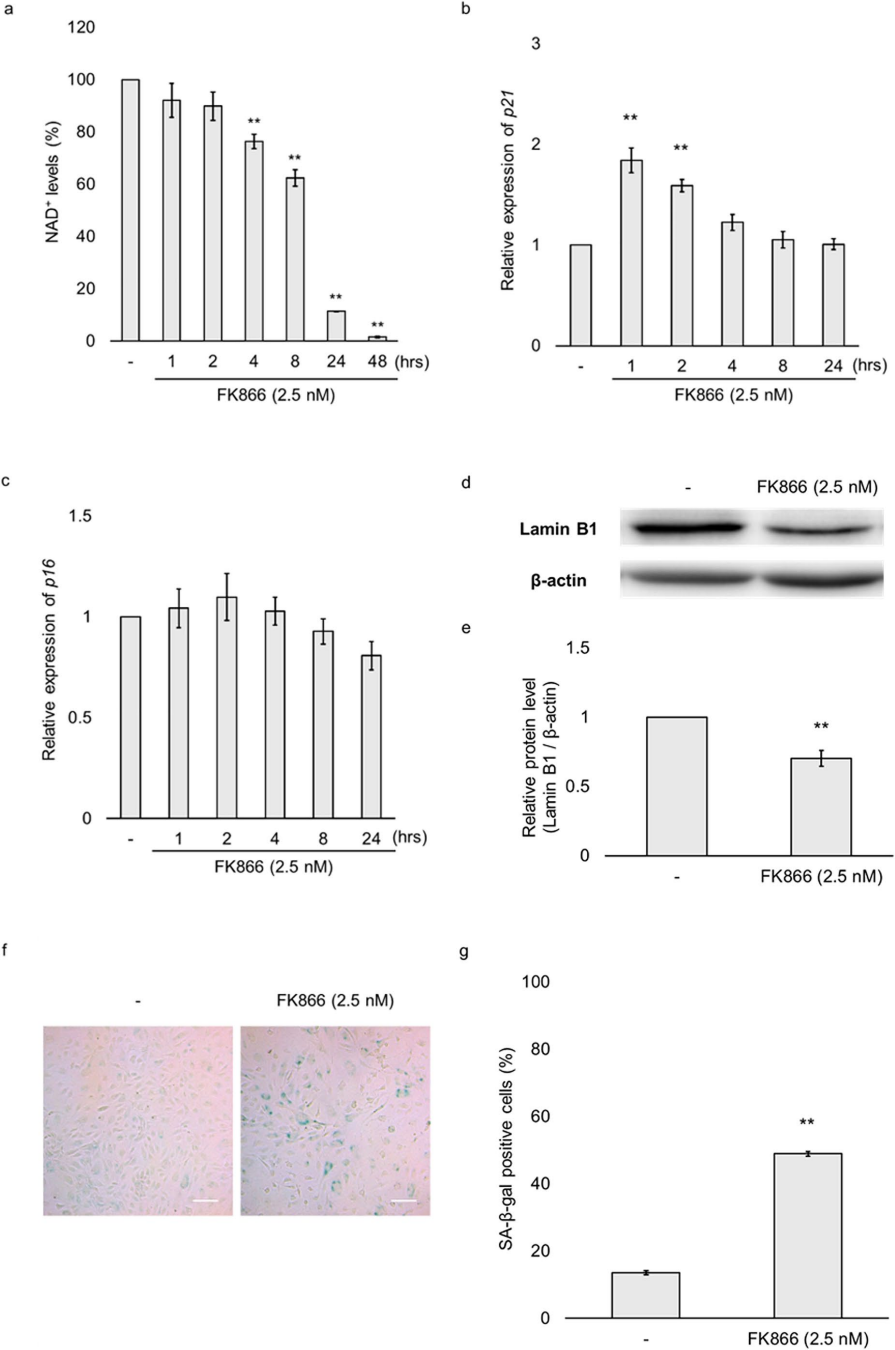
**Detection** **of intracellular** **nicotinamide adenine** **dinucleotide** **(NAD** ^**+**^ **) levels and cellular senescence markers in FK866‐treated human umbilical vein endothelial cells (HUVECs).** HUVECs were treated for the indicated hours with FK866 at a concentration of 2.5 nM. (a) Cellular NAD^+^ levels were determined using an NAD^+^/NADH assay kit. (b) The expression levels of *p21* mRNA were examined using qPCR. (c) The expression levels of *p16* mRNA were examined using qPCR. (d) Cells treated with FK866 for 48 h were analysed by immunoblotting using antibodies specific for Lamin B1. β‐actin was used as the loading control protein. (e) Quantification of Lamin B1 protein expression normalised to β‐actin. (f) Senescence‐associated β‐galactosidase (SA‐β‐gal) activity in FK866‐treated cells (48 h) was analysed using a Senescence Detection Kit. (g) The percentage of SA‐β‐gal‐positive cells. Scale bar: 50 μm. Data are presented as the mean ± standard error of the mean. **p < 0.01 compared to the untreated group.

We first confirmed that the NAMPT inhibitor FK866 effectively reduces intracellular NAD^+^ levels in HUVECs. Treatment with 2.5 nM FK866 resulted in a time‐dependent decrease in NAD^+^ levels, which became statistically significant after 4 h and declined to 1.5% of baseline within 48 h (Figure 1a). To determine whether FK866 induced cellular senescence, we assessed the expression of p21 and p16 mRNA. FK866 treatment significantly increased p21 mRNA expression after 1 and 2 h (Figure 1b). On the other hand, p16 mRNA expression was not induced within 48 h by FK866 treatment (Figure 1c). Subsequently, the expressions of Lamin B1 and SA‐β‐gal were investigated. FK866 treatment for 48 h significantly decreased Lamin B1 protein expression (Figure 1d,e) and significantly increased the percentage of SA‐β‐gal‐positive cells in HUVECs (Figure 1f,g). These results suggest that 2.5 nM FK866 treatment induces a transition to a senescence‐like phenotype.

### 3.2 FK866 Treatment Suppresses Proliferation Without a Detectable Increase in Cell Death in HUVECs

**FIGURE 2:**
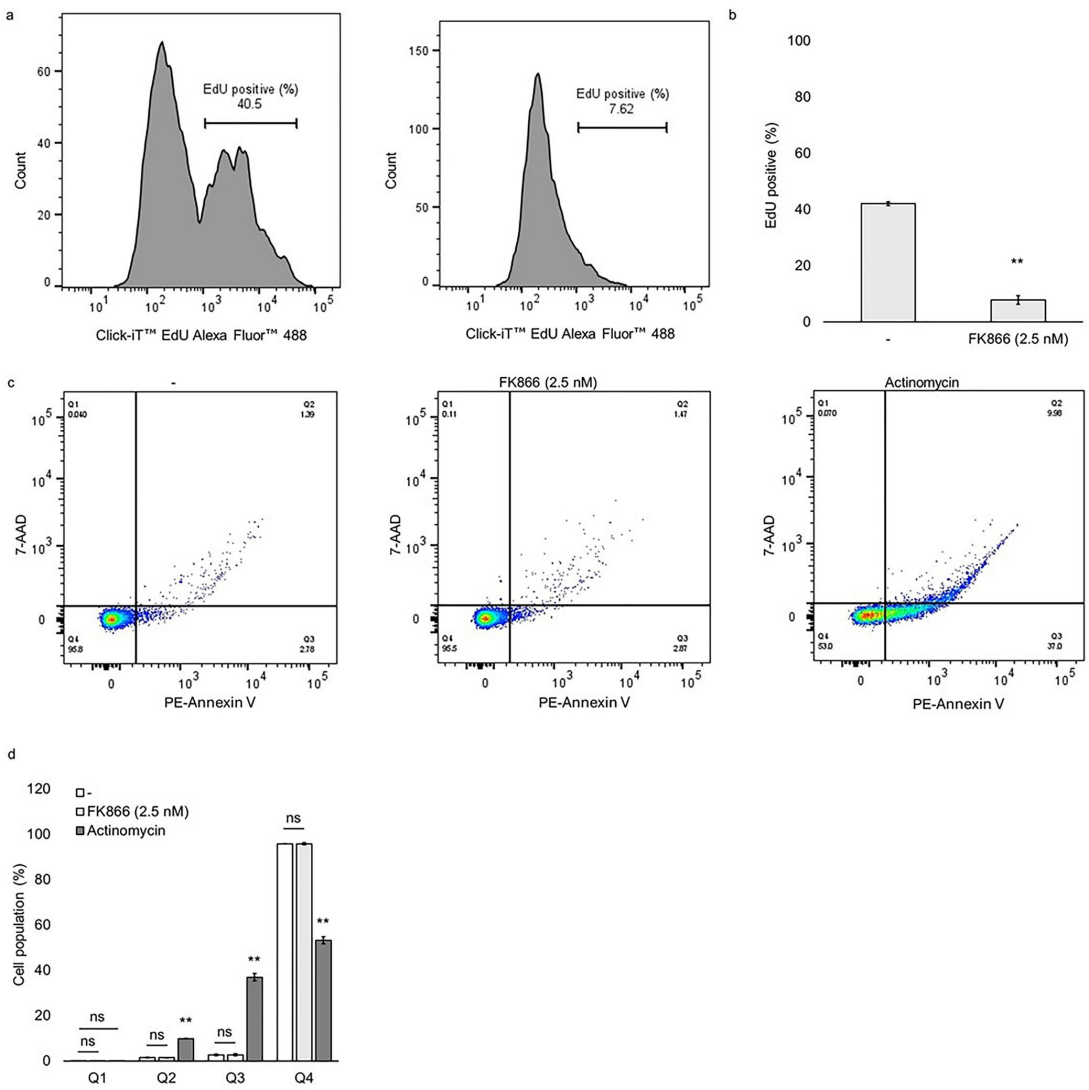
**Detection of EdU‐positive, apoptotic and necrotic cell populations in FK866‐treated HUVECs.** HUVECs were incubated with 2.5 nM FK866 for 48 h. (a) After FK866 treatment, cells were incubated with 10 μM EdU for 2 h. EdU incorporation was detected using the Click‐iT EdU Alexa Fluor 488 Flow Cytometry Assay Kit. The x‐axis indicates Alexa Fluor 488 fluorescence intensity (EdU incorporation), and the y‐axis represents cell count. (b) The percentage of EdU‐positive cells. (c) After FK866 treatment, cells were stained with PE‐Annexin V and 7‐AAD. Flow cytometric analysis was performed to distinguish different cell populations. Representative dot plots are shown, with PE‐Annexin V fluorescence on the x‐axis and 7‐AAD fluorescence on the y‐axis. Cell populations were classified as follows: necrotic (Q1; Annexin V^−^/7‐AAD^+^), late apoptotic (Q2; Annexin V^+^/7‐AAD^+^), early apoptotic (Q3; Annexin V^+^/7‐AAD^−^) and viable (Q4; Annexin V^−^/7‐AAD^−^) populations. (d) The percentages of cells in each population. Data are presented as the mean ± standard error of the mean. **p < 0.01 compared to the untreated group. Abbreviation: ns, not significant.

We performed an EdU incorporation assay to evaluate the effect of 2.5 nM FK866 on HUVEC proliferation using flow cytometry. The proportion of EdU‐positive cells was significantly decreased from 42.1% in the control group to 7.8% in the FK866‐treated group (Figure 2a,b). Furthermore, to assess cell death, PE‐Annexin V and 7‐AAD staining were performed. Cells were classified into necrotic (Q1; Annexin V^−^/7‐AAD^+^), late apoptotic (Q2; Annexin V^+^/7‐AAD^+^), early apoptotic (Q3; Annexin V^+^/7‐AAD^−^) and viable (Q4; Annexin V^−^/7‐AAD^−^) populations. In HUVECs treated with 2.5 nM FK866 for 48 h, there were no significant differences in the Q1, Q2, Q3 and Q4 populations compared to the untreated cell group. On the other hand, in the cell group treated with actinomycin for 24 h, the Q2 and Q3 (late‐stage and early‐stage apoptotic cells) populations were significantly increased, and the Q4 (viable) population was significantly decreased compared to the untreated cell group (Figure 2c,d). EdU incorporation analysis demonstrated a significant reduction in S‐phase DNA synthesis in FK866‐treated cells, indicating suppressed proliferation. PE‐Annexin V/7‐AAD staining showed no significant increase in apoptotic or necrotic populations. These findings indicate that FK866 treatment suppresses proliferation and does not significantly increase apoptotic or necrotic cell populations under the present experimental conditions.

### 3.3 FK866 Treatment Increases PGI_2_ and TXA_2_ Production by Upregulating COX2 Protein Expression in HUVECs

**FIGURE 3:**
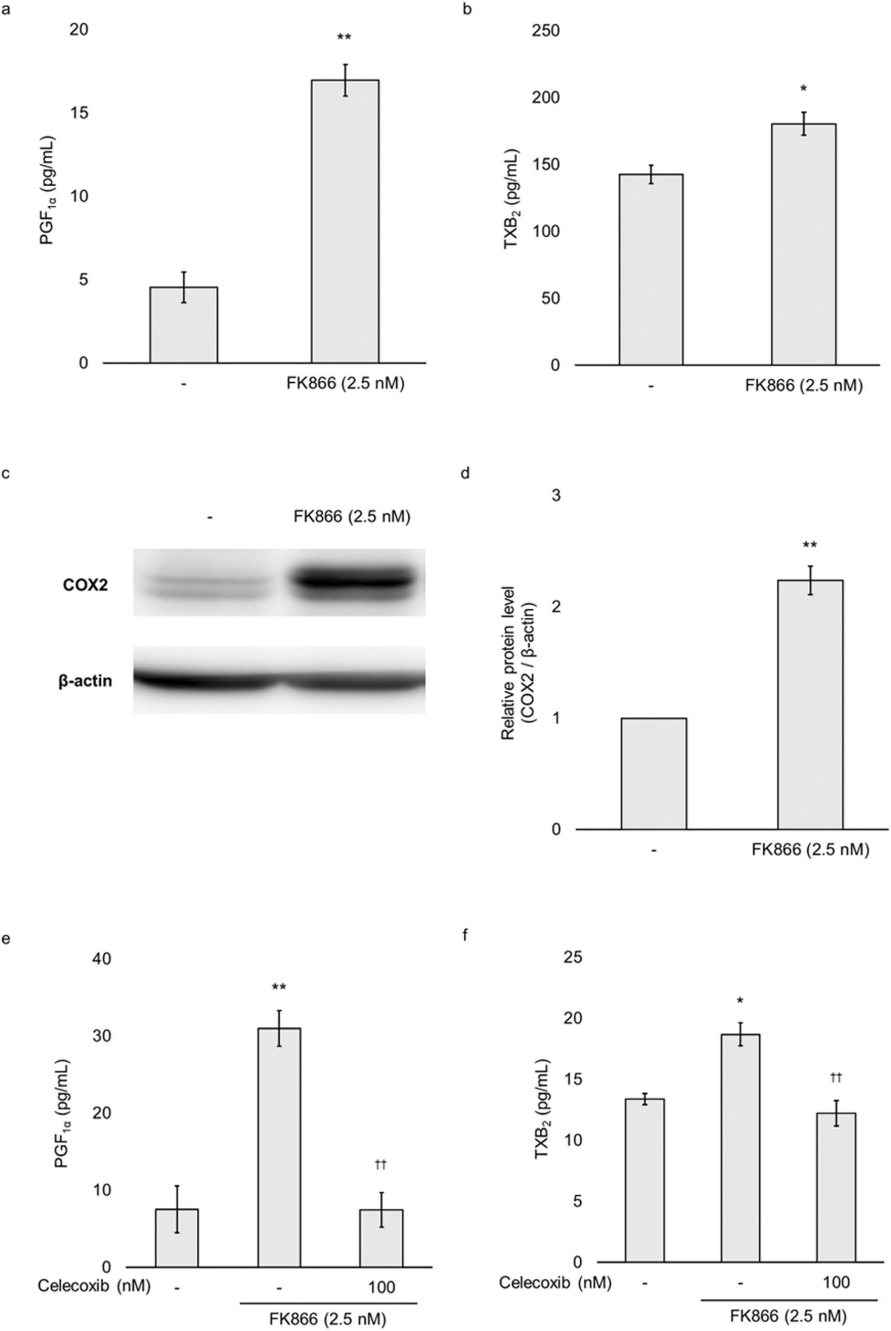
**Effect of 48 h FK866 treatment on the arachidonic acid cascade in HUVECs.** (a and b) HUVECs were incubated with 2.5 nM FK866 for 48 h. Prostaglandin F_1α_ (PGF_1α_) and thromboxane B_2_ (TXB_2_) concentrations in the cell supernatants collected 24 h after replacement with fresh medium were measured using ELISA. (c) Cells treated with FK866 for 48 h were analysed by immunoblotting using antibodies specific for cyclooxygenase2 (COX2). β‐actin was used as the loading control protein. (d) Quantification of COX2 protein expression normalised to β‐actin. (e and f) HUVECs were treated with FK866 for 48 h, with 100 nM celecoxib added for the final 24 h. PGF_1α_ and TXB_2_ concentrations in the cell supernatants collected 24 h after replacement with fresh medium were measured using ELISA. Data are presented as the mean ± standard error of the mean. *p < 0.05; **p < 0.01 compared to the untreated group. ^††^ *p* < 0.01 compared to the FK866‐treated group.

To assess whether FK866 affects prostanoid production in HUVECs, we measured the levels of PGI_2_ and TXA_2_. Because PGI_2_ and TXA_2_ are unstable under physiological conditions and rapidly degrade, we quantified their stable metabolites, PGF_1α_ and TXB_2_ respectively. After 48 h of FK866 treatment, the levels of PGF_1α_ and TXB_2_ were significantly elevated in the culture supernatants (Figure 3a,b). COX2 protein expression was also significantly upregulated under the same conditions (Figure 3c,d). Furthermore, the COX2 inhibitor celecoxib blocked FK866‐induced production of PGF_1α_ and TXB_2_ (Figure 3e,f). These results indicate that FK866 enhances PGI_2_ and TXA_2_ production by upregulating COX2 protein expression in HUVECs.

### 3.4 FK866 Treatment Increases COX2 Protein Expression by Activating the Stress‐Responsive p38 MAPK Pathway in HUVECs

**FIGURE 4:**
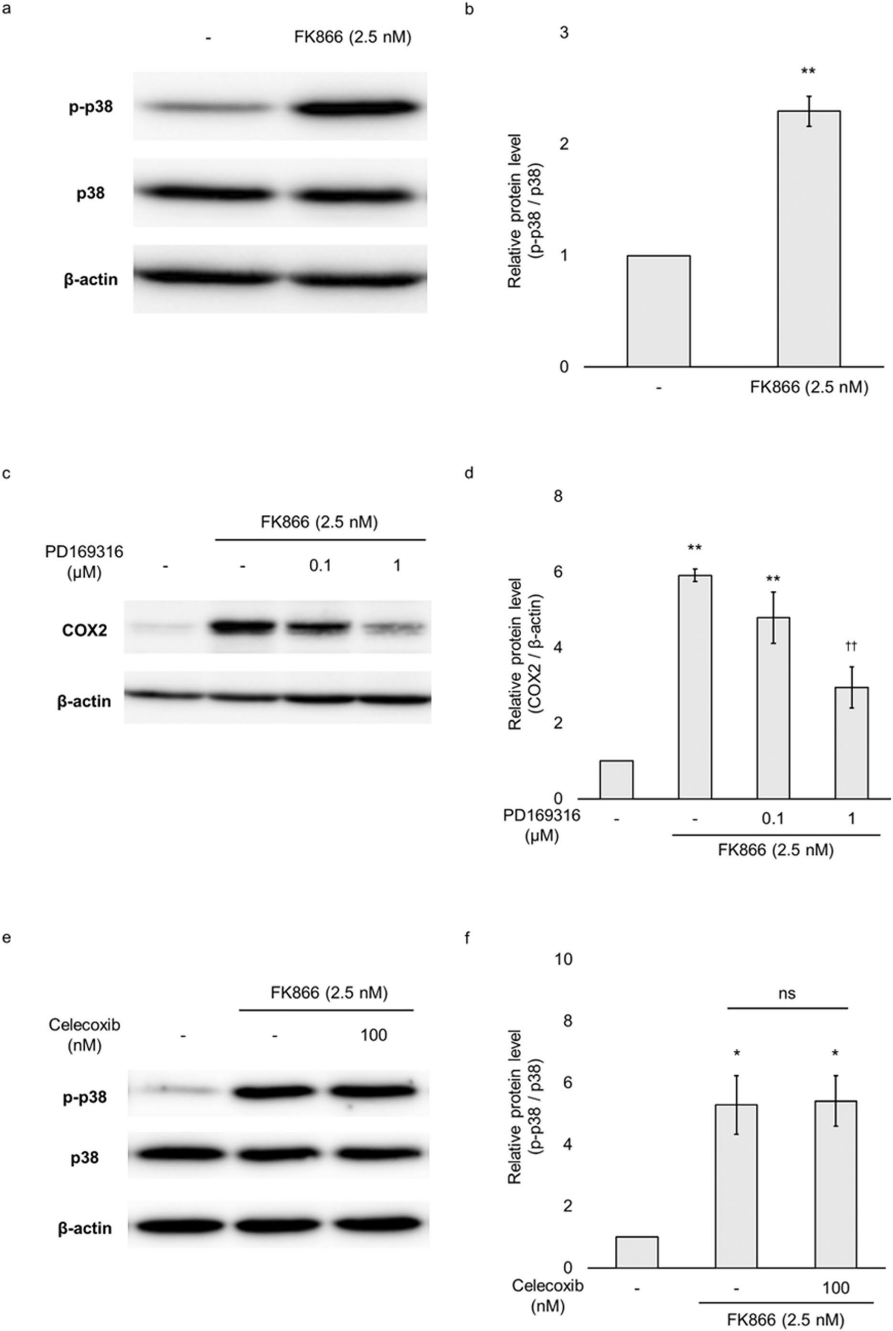
**Detection of mitogen‐activated protein kinase (MAPK) p38 protein as a mediator of COX2 upregulation in HUVECs treated with FK866 for 48 h.** (a) HUVECs incubated with 2.5 nM FK866 for 48 h were analysed by immunoblotting using antibodies specific for phosphorylated p38 and total p38. β‐actin was used as the loading control protein. (b) Quantification of p‐p38 protein expression normalised to total p38. (c) HUVECs were treated with FK866 for 48 h, with PD169316 added at the indicated concentrations for the final 24 h. The cells were analysed by immunoblotting using antibodies specific for COX2. β‐actin was used as the loading control protein. (d) Quantification of COX2 protein expression normalised to β‐actin. (e) HUVECs were treated with FK866 for 48 h, with 100 nM celecoxib added for the final 24 h. Cells were analysed by immunoblotting using antibodies specific for p‐p38 and total p38. β‐actin was used as the loading control protein. (f) Quantification of p‐p38 protein expression normalised to total p38. Data are presented as the mean ± standard error of the mean. *p < 0.05; **p < 0.01 compared to the untreated group. ^††^ *p* < 0.01 compared to the FK866‐treated group. Abbreviation: ns, not significant.

To investigate the signalling mechanism underlying COX2 upregulation by FK866, we investigated the activation of transcriptional regulators involved in COX2 expression. FK866 treatment did not induce phosphorylation of p65, the transcriptionally active subunit of NF‐κB (Figure S1a). Among the three MAPK pathways tested—ERK, JNK and p38—only p38 showed increased phosphorylation in response to FK866, whereas ERK and JNK phosphorylation levels remained unchanged (Figures S1b,c and 4a,b). Furthermore, the addition of the p38 inhibitor PD169316 after FK866 treatment significantly suppressed FK866‐induced COX2 expression (Figure 4c,d). BIRB796 and PH‐797804, p38 inhibitors with structural differences from PD169316, similarly significantly suppressed the FK866‐induced COX2 expression (Figure S2a–d). In HUVECs in which p38 expression was suppressed to 29% (vs. FK866‐treated si‐control group) using p38α‐targeted small interfering RNA (siRNA), 48 h FK866 treatment did not induce a significant increase in COX2 expression (Figure S2e–g). In contrast, celecoxib did not affect p38 phosphorylation (Figure 4e,f). These results suggest that FK866 induced COX2 expression in HUVECs via the p38 MAPK pathway.

### 3.5 Restoration of Intracellular NAD ^+^ Levels by NMN Suppresses FK866‐Induced Cellular Senescence in HUVECs

**FIGURE 5:**
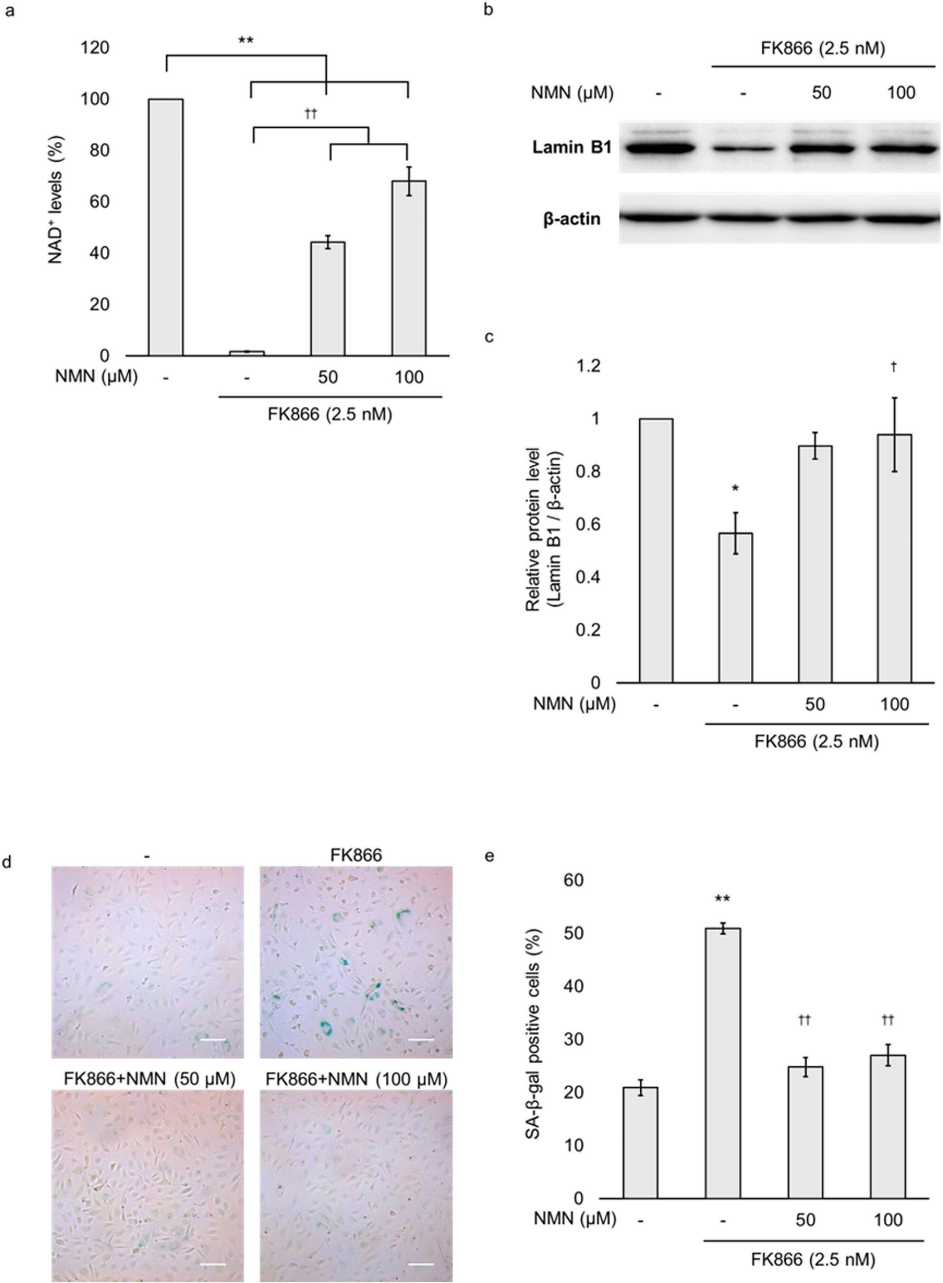
**Effect of nicotinamide mononucleotide (NMN)** **on FK866‐induced decrease in intracellular** **NAD** ^**+**^ **levels and cellular senescence in HUVECs.** HUVECs were treated with 2.5 nM FK866 for 48 h, with or without NMN at the indicated concentrations during the final 24 h. (a) Cellular NAD^+^ levels were determined using an NAD^+^/NADH assay kit. (b) Cells were analysed by immunoblotting with antibodies specific for Lamin B1. β‐actin was used as the loading control protein. (c) Quantification of Lamin B1 protein expression normalised to β‐actin. (d) Senescence‐associated β‐galactosidase (SA‐β‐gal) activity was analysed using a Senescence Detection Kit. (e) The percentage of SA‐β‐gal‐positive cells. Scale bar: 50 μm. Data are presented as the mean ± standard error of the mean. *p < 0.05; **p < 0.01 compared to the untreated group. ^†^ *p* < 0.05; ^††^ *p* < 0.01 compared to the FK866‐treated group.

To investigate whether NAD^+^ depletion directly induces cellular senescence in HUVECs, we assessed whether supplementation with the NAD^+^ precursor NMN could reverse FK866‐induced cellular senescence. HUVECs were treated with FK866 for 48 h, and NMN (50 or 100 μM) was co‐administered during the final 24 h. Under these conditions, intracellular NAD^+^ levels were significantly restored to 44% and 68% of baseline levels with 50 and 100 μM NMN respectively (Figure 5a). NMN treatment significantly inhibited the FK866‐induced reduction in Lamin B1 protein expression at 100 μM (Figure 5b,c). Additionally, the proportion of SA‐β‐gal‐positive cells was significantly decreased by co‐treatment with 50 and 100 μM NMN (Figure 5d,e). These results suggest that the cellular senescence observed in FK866‐treated HUVECs was mediated, at least in part, by intracellular NAD^+^ depletion.

### 3.6 Restoration of Intracellular NAD ^+^ Levels by NMN Suppresses FK866‐Induced PGI_2_ and TXA_2_ Production via the p38/COX2 Pathway in HUVECs

**FIGURE 6:**
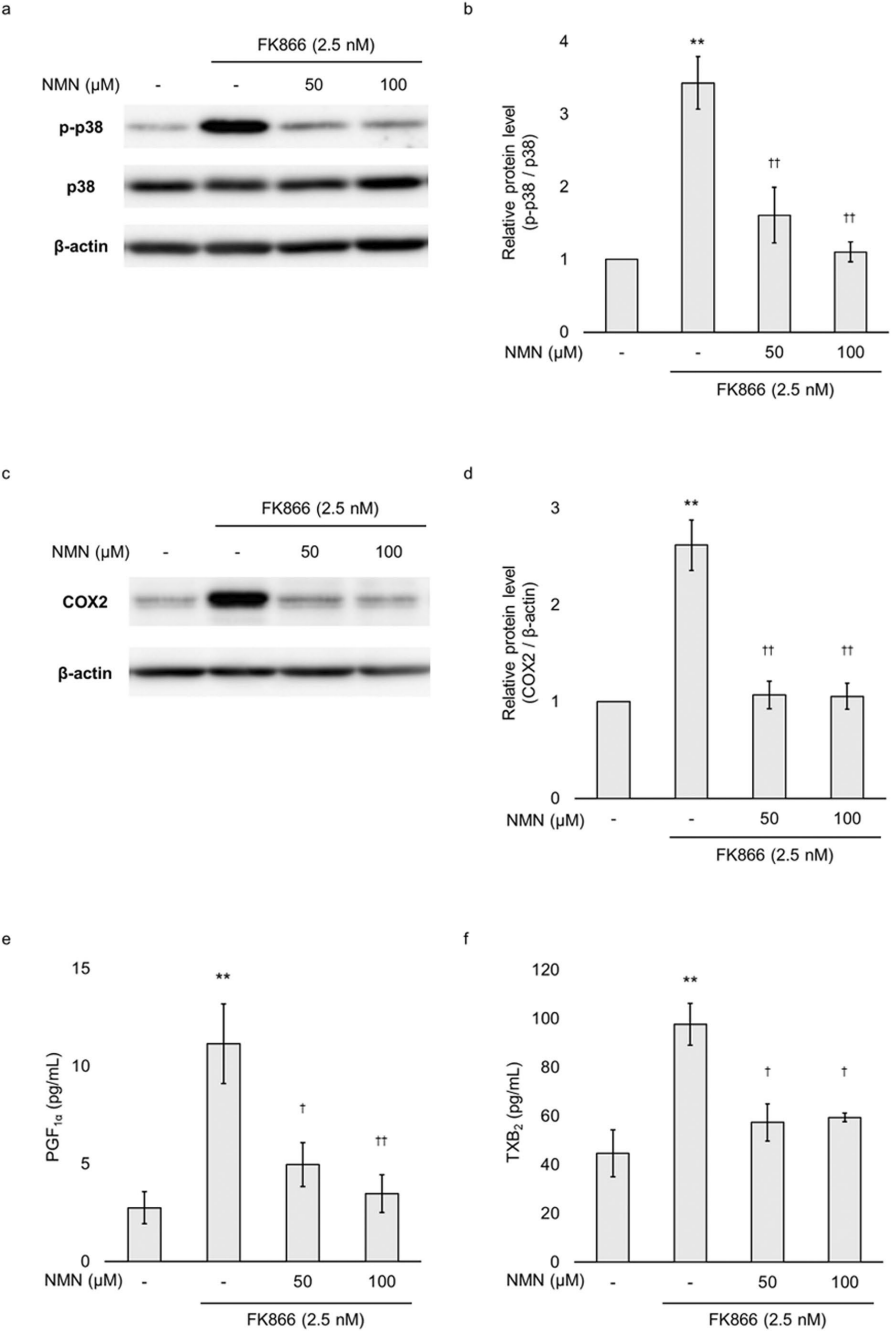
**Effect of NMN on FK866‐induced changes in p38/COX2 signal activation and prostanoid production.** HUVECs were treated with 2.5 nM FK866 for 48 h, with or without NMN added at the indicated concentrations during the final 24 h. (a) Cells were analysed by immunoblotting with antibodies specific for phosphorylated p38 and total p38. β‐actin was used as the loading control protein. (b) Quantification of p‐p38 protein expression normalised to total p38. (c) Cells were analysed by immunoblotting using antibodies specific for COX2. β‐actin was used as the loading control protein. (d) Quantification of COX2 protein expression normalised to β‐actin. (e and f) PGF_1α_ and TXB_2_ concentrations in the cell supernatants collected 24 h after replacement with fresh medium were measured using ELISA. Data are presented as the mean ± standard error of the mean. **p < 0.01 compared to the untreated group. ^†^ *p* < 0.05; ^††^ *p* < 0.01 compared to the FK866‐treated group.

We next examined whether the FK866‐induced activation in the p38/COX2 signalling pathway, and PGI_2_ and TXA_2_ production were attributable to NAD^+^ depletion. Co‐treatment with NMN during the final 24 h of FK866 exposure significantly suppressed the increase in phosphorylated p38 and COX2 expression at both 50 and 100 μM (Figure 6a–d). In parallel, NMN significantly inhibited the FK866‐induced elevation in PGF_1α_ and TXB_2_ production (Figure 6e,f). These results indicate that the FK866‐induced activation of the p38/COX2 pathway and increase in prostanoid production were driven by NAD^+^ depletion in HUVECs.

## 4 Discussion

Abnormalities in prostanoid production are thought to contribute to the development of CVD. Although age‐associated NAD^+^ decline has been implicated in vascular dysfunction, it remains unclear whether intracellular NAD^+^ depletion itself is sufficient to promote prostanoid production. In our study, we demonstrated that NAD^+^ depletion in HUVECs significantly enhanced PGF_1α_ and TXB_2_ production via the p38/COX2 signalling pathway.

FK866 treatment reduced NAD^+^ levels to 1.5% of baseline in HUVECs, a more severe decrease compared to that associated with physiological ageing. Total NAD levels were reduced to 54.8% in HUVECs treated with NAMPT‐targeting siRNA, but no increase in the percentage of SA‐β‐gal‐positive cells was observed (Figure S3a–c). COX2 protein expression was not increased in si‐NAMPT‐treated HUVECs (Figure S3d,e). NAMPT siRNA led to approximately 70% reduction in NAMPT protein expression in HUVECs (Figure S3d,f). A rapid decrease in intracellular NAD^+^ by approximately 80% has been reported to induce the accumulation of DNA damage and may induce cellular senescence [22]. Furthermore, in an inflammatory bowel disease model, mice, severe NAD depletion has been reported to enhance mRNA levels of inflammatory cytokines in the intestinal epithelium [23]. Therefore, not slight but severe decreased NAD levels may drive HUVECs towards a senescence‐like phenotype and increased COX2‐derived prostanoid production.

Although our study focused on PGs and TXs produced through the arachidonic acid‐COX pathway, other lipid mediators such as leukotrienes (LTs) and lipoxins (LXs), synthesised through the 5‐ and 15‐lipoxygenase (LOX) pathways, respectively, also play critical roles in cardiovascular function. LTs are produced primarily in immune cells, such as macrophages, and are involved in allergic and inflammatory responses, particularly in the progression of arteriosclerosis [2]. In contrast, LXs are known to resolve inflammation and suppress neutrophil migration, and have been shown to exert protective effects in CVD [24]. Because LT levels increase and LX levels decrease with ageing [25, 26, 27, 28], both are considered potential therapeutic targets in age‐related cardiovascular disorders. In addition, high expression of an NAD^+^‐consuming enzyme, CD38, has been reported in aged macrophages, suggesting systemic NAD^+^ depletion in ageing tissues. However, few studies have investigated whether NAD^+^ depletion directly affects LOX expression or LT/LX biosynthesis. Although our findings were limited to endothelial cells, we observed that NAD^+^ depletion activated p38 MAPK and upregulated COX2 expression. Given that inflammatory pathways are often upregulated in NAD^+^‐depleted cells, it is plausible that 5‐LOX and 15‐LOX expressions may increase and decrease, respectively, in aged or NAD^+^‐deficient macrophages. Therefore, systemic supplementation with NAD^+^ precursors, such as NMN, is expected to return 5‐LOX and 15‐LOX expression to normal conditions in blood vessel macrophages. LTs and LXs act on vascular endothelial cells through specific receptors [29, 30], and antagonists of LT receptors have been shown to exert protective effects in vascular tissues, including the inhibition of atherosclerosis progression [31]. Future studies should assess whether NAD^+^‐depletion in endothelial cells alters LOX pathway enzymes or their receptor expressions.

In our NAD^+^ depletion model, FK866 did not induce p65 phosphorylation, suggesting that canonical NF‐κB activation was not prominently induced. Furthermore, we examined the phosphorylation of the three MAPK molecules and observed an increase in p38 phosphorylation (Figures S1b,c and 4a,b). Considering the results in Figures 4 and S2, it is suggested that FK866 increases COX2 expression via p38 activation. Determining the involvement of the NF‐κB pathway requires assessment of nuclear translocation and inhibition experiments. However, considering the results of p38 inhibitors and knockdown studies, FK866 increased COX2 expression via activating p38 MAPK signalling, leading to prostanoid production. This finding is consistent with previous research that chronic inflammation upregulates cytokines, such as TNF‐α and IL‐1β, which enhance arachidonic acid metabolism via p38 MAPK activation [2]. On the other hand, it has been reported that FK866 inhibits rather than induces p38 activation [32, 33, 34]. For example, it has been reported that FK866 treatment reduces lung injury in a pulmonary ischaemia–reperfusion injury model by suppressing the production of inflammatory cytokines via inhibiting the phosphorylation of p38 [32]. To the best of our knowledge, no prior reports have demonstrated that NAD^+^ depletion activates p38 MAPK in vascular endothelial cells. One possible explanation is that activation of NADPH oxidase during NAD^+^ depletion induces oxidative stress, which is known to activate p38 MAPK and contribute to endothelial dysfunction [35, 36]. Further studies are required to determine whether NAD^+^ depletion leads to oxidative stress and p38 activation in endothelial cells.

The functional role of prostanoids in vascular physiology is complex, with each species capable of exerting opposing or context‐dependent effects. PGI_2_ has been reported to act on both vasodilator IP receptors and vasoconstrictive thromboxane–prostanoid receptors. Depending on receptor distribution and activity in the vascular bed, either vasodilation or vasoconstriction may be predominant [37]. PGI_2_ and TXA_2_ are often co‐produced and act in a counter‐regulatory manner to maintain vascular tone. A disruption of this balance may lead to vascular pathologies, such as stroke or cerebral ischaemia/reperfusion injury. Therefore, maintaining prostanoid homeostasis is considered essential for cardiovascular health [38, 39]. In our NAD^+^ depletion‐induced HUVECs senescence model, both TXA_2_ and PGI_2_ production were significantly increased, leading to their concurrent increase. Notably, the expression of prostacyclin synthase and thromboxane synthase enzymes was not increased in FK866‐treated cells (data not shown), suggesting that prostanoid production concurrently increased primarily from the upregulation of upstream COX2. This is consistent with our observation that celecoxib, a selective COX2 inhibitor, suppressed both PGF_1α_ and TXB_2_ production (Figure 2e,f), indicating that COX2 activity drove the observed prostanoid elevation. These findings support a mechanistic link between NAD^+^ depletion, COX2 induction and concurrent prostanoid increase. Although COX2 inhibitors may have the potential to treat CVD, long‐term use of selective COX2 inhibitors is associated with an increased risk of adverse cardiovascular events, and their clinical utility remains limited [40]. Previous studies have shown that NMN suppresses NAD^+^ depletion‐induced cytoskeletal disruption and oxidative stress in vascular endothelial cells, suggesting the potential of NMN for CVD treatment and prevention [41, 42].

In our study, NMN not only restored intracellular NAD^+^ levels but also normalised COX2 expression and mitigated the concurrent increase in prostanoid production. These findings provide a novel perspective: NAD^+^ replenishment may be an effective strategy to regulate COX2‐driven inflammation and vascular dysfunction in ageing. However, current data on NMN safety and efficacy are limited to animal models, with studies generally lasting up to 6 months [43]. Although further clinical trials are required, our results support the therapeutic potential of NMN in mitigating age‐associated vascular dysfunction through NAD^+^ homeostasis.

This study has several limitations. It was limited to an in vitro HUVEC model, which may not fully represent in vivo vascular conditions. NAD^+^ depletion was induced pharmacologically with FK866, which may not reflect the gradual age‐related decline. The effects of long‐term NAD^+^ precursor supplementation and other lipid mediators, such as LTs and LXs, were not examined. Furthermore, to understand vascular endothelial function from a more multifaceted perspective, functional readouts such as vasorelaxation assays or platelet aggregation relevance are necessary. Future studies should validate these results in vivo and assess the long‐term efficacy and safety of NAD^+^ precursors.

## Author Contributions

**Natsuko Kitajima:** conceptualization, methodology, formal analysis, investigation, writing – original draft, writing – review and editing. **Kentaro Tsuji:** methodology, writing – review and editing. **Takeshi Katayoshi:** methodology. **Takahisa Nakajo:** conceptualization, methodology, formal analysis, investigation, writing – original draft, writing – review and editing, supervision.

## Funding

The authors have nothing to report.

## Ethics Statement

The cell lines used in the present study are commercially available, collected from donors with informed consent under the supplier's ethical approval. No further ethics approval was required.

## Consent

The authors have nothing to report.

## Conflicts of Interest

All authors are employees of DHC Corporation. The authors declare no conflicts of interest.

## Supporting information


**Data S1:** SUPPORTING MATERIALS AND METHODS.
**Figure S1:** **Detection of NF‐κB and MAPK activities.** Human umbilical vein endothelial cells (HUVECs) were treated with FK866 at a concentration of 2.5 nM for 48 h. (a) Immunoblotting using antibodies targeting p‐p65 and p65. (b) Immunoblotting using antibodies targeting p‐ERK and ERK. (c) Immunoblotting using antibodies targeting p‐JNK and JNK. β‐actin was used as a loading control protein.
**Figure S2:** **Detection of mitogen‐activated protein kinase (MAPK) p38 protein as a mediator of COX2 upregulation in HUVECs treated with FK866 for 48 h.** (a) HUVECs were treated with FK866 for 48 h, with BIRB796 added at the indicated concentrations for the final 24 h. The cells were analysed by immunoblotting using antibodies specific for COX2. β‐actin was used as the loading control protein. (b) Quantification of COX2 protein expression normalised to β‐actin. (c) Cells were treated with FK866 for 48 h, with PH‐797804 added at the indicated concentrations for the final 24 h. The cells were analysed by immunoblotting using antibodies specific for COX2. β‐actin was used as the loading control protein. (d) Quantification of COX2 protein expression normalised to β‐actin. Data are presented as the mean ± standard error of the mean. **p < 0.01 compared to the untreated group. ^†^ *p* < 0.05; ^††^ *p* < 0.01 compared to the FK866‐treated group. (e) Cells were cultured with the indicated siRNA (150 ng/mL), 3 μL of HiPerFect Transfection Reagent and 2.5 nM FK866 for 48 h. Cells were analysed by immunoblotting using antibodies specific for COX2 and total p38. β‐actin was used as the loading control protein. (f) Quantification of COX2 protein expression normalised to total β‐actin. (g) Quantification of total p38 protein expression normalised to total β‐actin. Data are presented as the mean ± standard error of the mean. **p < 0.01 compared to the si‐control group. ^††^ *p* < 0.01 compared to the si‐control with the FK866‐treated group. Abbreviation: ns, not significant.
**Figure S3: Effect of NAMPT protein knockdown on cellular senescence and COX2 protein expression** HUVECs were transfected with the indicated siRNA (150 ng/mL) using 3 μL of HiPerFect Transfection Reagent for 72 h. (a) Cellular total NAD levels were determined using an NAD^+^/NADH assay kit. (b) Senescence‐associated β‐galactosidase (SA‐β‐gal) activity in cells was analysed using a Senescence Detection Kit. (c) The percentage of SA‐β‐gal‐positive cells. Scale bar: 50 μm. (d) Cells were analysed by immunoblotting using antibodies specific for COX2 and NAMPT. β‐actin was used as the loading control protein. (e) Quantification of COX2 protein expression normalised to total β‐actin. (f) Quantification of NAMPT protein expression normalised to total β‐actin. Data are presented as the mean ± standard error of the mean. **p < 0.01 compared to the si‐control group. Abbreviation: ns, not significant.

## Data Availability

The data that support the findings of this study are available from the corresponding author upon reasonable request.
