# Supplementary material for: Intracellular NAD + Depletion Increases Prostanoid Production via p38/COX2 Signalling in FK866‐Induced Senescent Human Umbilical Vein Endothelial Cells
Source: J Cell Mol Med. 2026 May 20;30(10):e71187. doi: 10.1111/jcmm.71187 (PMC13240423; doi:10.1111/jcmm.71187)
Supplement: Supplementary file 1 — Data S1: SUPPORTING MATERIALS AND METHODS. Figure S1: Detection of NF‐κB and MAPK activities. Human umbilical vein endothelial cells (HUVECs) were treated with FK866 at a concentration of 2.5 nM for 48 h. (a) Immunoblotting using antibodies targeting p‐p65 and p65. (b) Immunoblotting using antibodies targeting p‐ERK and ERK. (c) Immunoblotting using antibodies targeting p‐JNK and JNK. β‐actin was used as a loading control protein. Figure S2: Detection of mitogen‐activated protein kinase (MAPK) p38 protein as a mediator of COX2 upregulation in HUVECs treated with FK866 for 48 h. (a) HUVECs were treated with FK866 for 48 h, with BIRB796 added at the indicated concentrations for the final 24 h. The cells were analysed by immunoblotting using antibodies specific for COX2. β‐actin was used as the loading control protein. (b) Quantification of COX2 protein expression normalised to β‐actin. (c) Cells were treated with FK866 for 48 h, with PH‐797804 added at the indicated concentrations for the final 24 h. The cells were analysed by immunoblotting using antibodies specific for COX2. β‐actin was used as the loading control protein. (d) Quantification of COX2 protein expression normalised to β‐actin. Data are presented as the mean ± standard error of the mean. **p < 0.01 compared to the untreated group. † p < 0.05; †† p < 0.01 compared to the FK866‐treated group. (e) Cells were cultured with the indicated siRNA (150 ng/mL), 3 μL of HiPerFect Transfection Reagent and 2.5 nM FK866 for 48 h. Cells were analysed by immunoblotting using antibodies specific for COX2 and total p38. β‐actin was used as the loading control protein. (f) Quantification of COX2 protein expression normalised to total β‐actin. (g) Quantification of total p38 protein expression normalised to total β‐actin. Data are presented as the mean ± standard error of the mean. **p < 0.01 compared to the si‐control group. †† p < 0.01 compared to the si‐control with the FK866‐treated group. Abbreviation: ns, not signifi [file JCMM-30-e71187-s001.docx]

***Supplementary Information*** ***for***

**Intracellular NAD^+^ depletion increases prostanoid production via p38/COX2 signaling in FK866-induced senescent human umbilical vein endothelial cells**

Natsuko Kitajima, Takahisa Nakajo, Takeshi Katayoshi, and Kentaro Tsuji*

*DHC Corporation Laboratories, Division 2, 2-42 Hamada, Mihama-ku, Chiba 261-0025, Japan*

***Corresponding author:**

Kentaro Tsuji

E-mail address: [ktsuji@dhc.co.jp](mailto:ktsuji@dhc.co.jp)

**SUPPLEMENTARY MATERIALS AND METHODS**

**Reagents**

Anti-phospho-NF-κB p65 (#3033, 1:5000), anti- NF-κB p65 (#8242, 1:5000), anti- Phospho-p44/42 MAPK (ERK1/2) (#4370, 1:5000), anti-p44/42 MAPK (ERK1/2) (#9102, 1:5000), anti-phospho-SAPK/JNK (#4668, 1:1000), anti-JNK2 (#9258, 1:1000), and anti-NAMPT (#86634, 1:5000) antibodies were purchased from Cell Signaling Technology (Danvers, MA, USA). BIRB796 and PH-797804 were purchased from Selleck Chemicals (Houston, TX, USA). All other reagents used were of the highest commercially available grade.

**RNA silencing**

HUVECs were transfected with ON-TARGETplus p38α siRNA (No. L-003512-00-0005, Dharmacon), ON-TARGETplus Non-targeting Pool (No. D-001810-10-20, Dharmacon, Lafayette, CO), or ON-TARGETplus NAMPT siRNA (No. L-004581-00-0005, Dharmacon) using HiPerFect Transfection Reagent (Qiagen). In particular, cells were transfected with the indicated siRNA (150 ng/mL) using 3 μL of HiPerFect Transfection Reagent for 48 or 72 h.

**Statistical analysis**

Data are expressed as the mean ± standard error from at least three independent biological experiments. Statistical significance was evaluated using the Student’s *t*-test for two-group comparisons and one-way analysis of variance followed by the Tukey–Kramer test for multiple comparisons involving three or more groups, with Statcel3 software (OMS, Tokyo, Japan). A *p*-value < 0.05 was considered statistically significant.

**
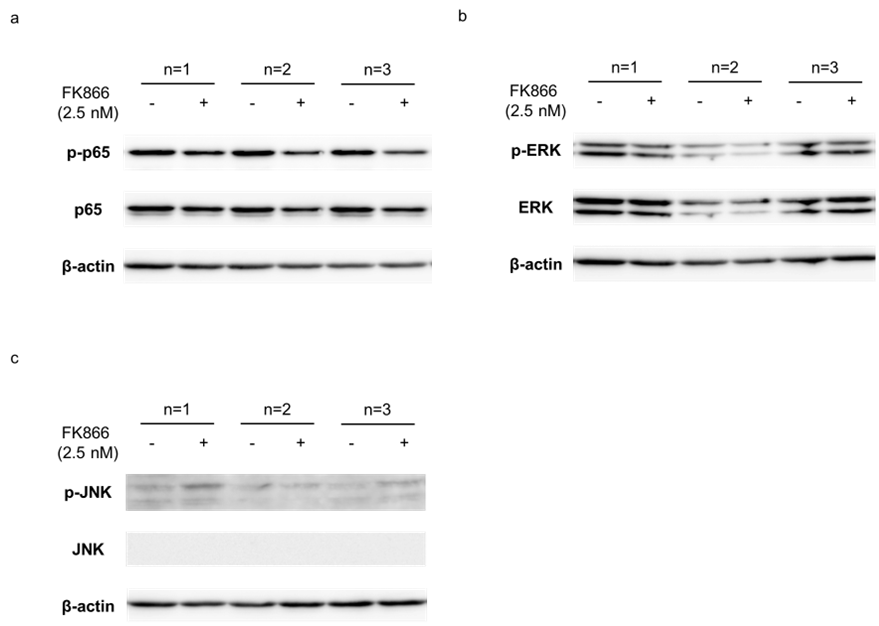
SUPPLEMENTARY FIGURES**

**Supplementary Fig. 1. Detection of NF-κB and MAPK activities.** Human umbilical vein endothelial cells (HUVECs) were treated with FK866 at a concentration of 2.5 nM for 48 h. (a) Immunoblotting using antibodies targeting p-p65 and p65. (b) Immunoblotting using antibodies targeting p-ERK and ERK. (c) Immunoblotting using antibodies targeting p-JNK and JNK. β-actin was used as a loading control protein.


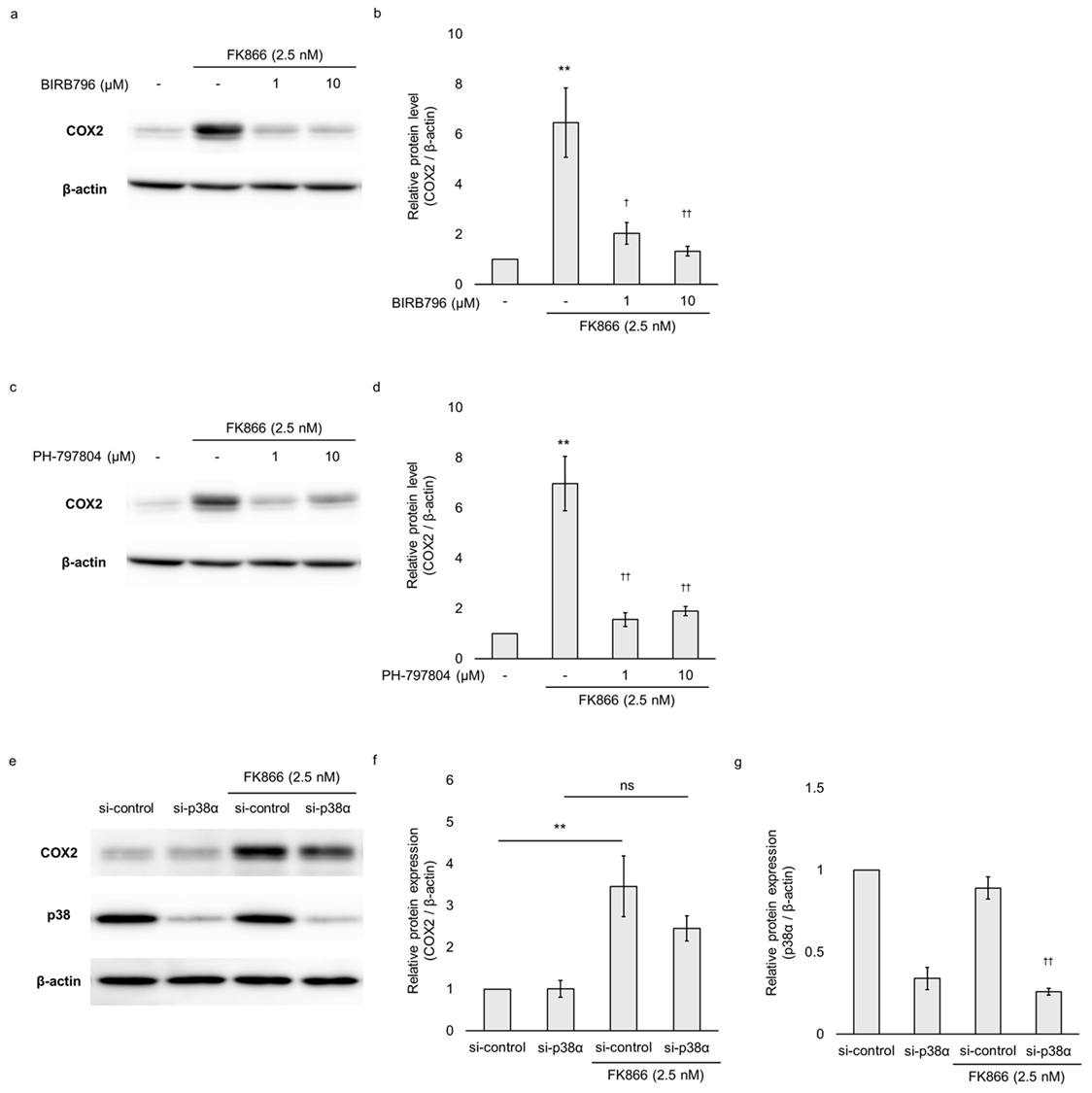


**Supplementary Fig. 2 Detection of mitogen-activated protein kinase (MAPK) p38 protein as a mediator of COX2 upregulation in HUVECs treated with FK866 for 48 h.** (a) HUVECs were treated with FK866 for 48 h, with BIRB796 added at the indicated concentrations for the final 24 h. The cells were analyzed by immunoblotting using antibodies specific for COX2. β-actin was used as the loading control protein. (b) Quantification of COX2 protein expression normalized to β-actin. (c) Cells were treated with FK866 for 48 h, with PH-797804 added at the indicated concentrations for the final 24 h. The cells were analyzed by immunoblotting using antibodies specific for COX2. β-actin was used as the loading control protein. (d) Quantification of COX2 protein expression normalized to β-actin. Data are presented as the mean ± standard error of the mean. ***p* < 0.01 compared to the untreated group. ^†^*p* < 0.05; ^††^*p* < 0.01 compared to the FK866 treated group. (e) Cells were cultured with indicated siRNA (150 ng/mL), 3 μL of HiPerFect Transfection Reagent, and 2.5 nM FK866 for 48 h. Cells were analyzed by immunoblotting using antibodies specific for COX2 and total p38. β-actin was used as the loading control protein. (f) Quantification of COX2 protein expression normalized to total β-actin. (g) Quantification of total p38 protein expression normalized to total β-actin. Data are presented as the mean ± standard error of the mean. ***p* < 0.01 compared to the si-control group. ^††^*p* < 0.01 compared to the si-control with FK866 treated group. ns, not significant

**
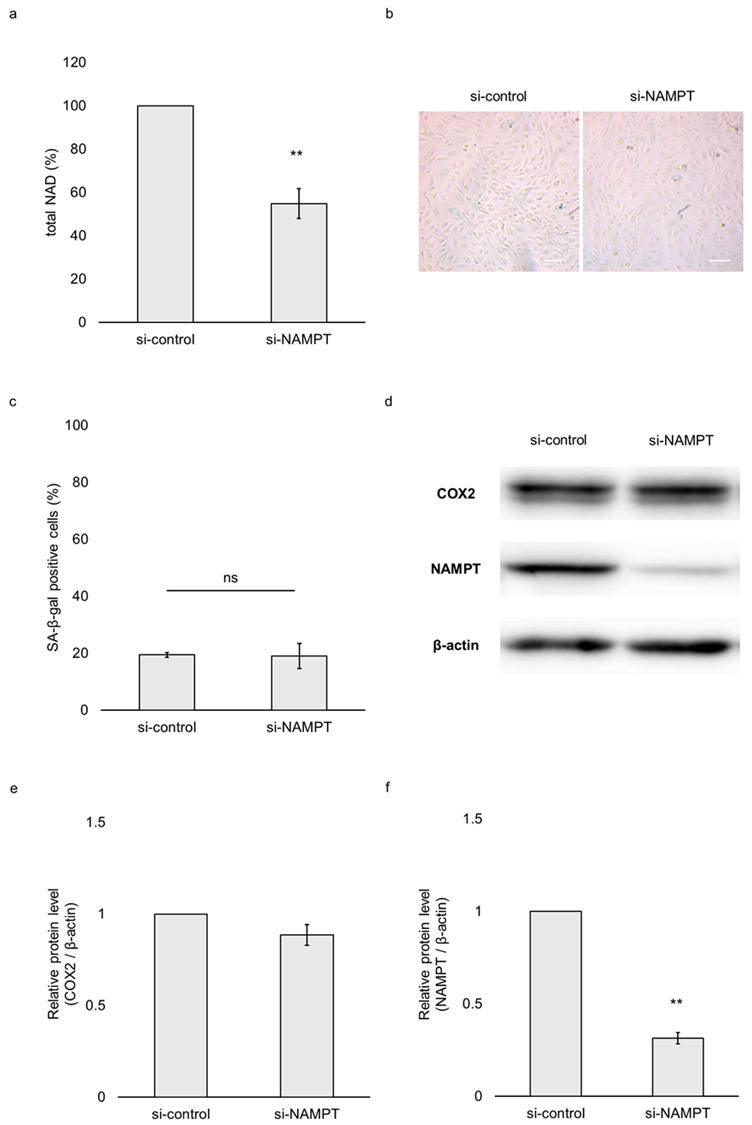
**

**Supplementary Fig. 3. Effect of NAMPT protein knock down on cellular senescence and COX2 protein expression.** HUVECs were transfected with the indicated siRNA (150 ng/mL) using 3 μL of HiPerFect Transfection Reagent for 72 h. (a) Cellular total NAD levels were determined using an NAD^+^/NADH assay kit. (b) Senescence-associated β-galactosidase (SA-β-gal) activity in cells was analyzed using a Senescence Detection Kit. (c) The percentage of SA-β-gal-positive cells. Scale bar: 50 μm. (d) Cells were analyzed by immunoblotting using antibodies specific for COX2 and NAMPT. β-actin was used as the loading control protein. (e) Quantification of COX2 protein expression normalized to total β-actin. (f) Quantification of NAMPT protein expression normalized to total β-actin. Data are presented as the mean ± standard error of the mean. ***p* < 0.01 compared to the si-control group. ns, not significant
